# The teaching and learning of health advocacy in an Australian medical school

**DOI:** 10.5116/ijme.5a4b.6a15

**Published:** 2018-01-31

**Authors:** Arabelle Douglas, Donna Mak, Caroline Bulsara, David Macey, Indira Samarawickrema

**Affiliations:** 1 School of Medicine, University of Notre Dame Australia, Australia; 2 Population and Preventive Health Domain, School of Medicine, University of Notre Dame Australia, Australia; 3 School of Nursing & Midwifery, University of Notre Dame Australia, Australia; 4 School of Veterinary and Life Sciences, Murdoch University, Perth, Australia

**Keywords:** Teaching learning health advocacy, medical education, Australia

## Abstract

**Objectives:**

To determine if medical graduates from an Australian university are educated and skilled in health advocacy for their future practice with patients and the wider community.

**Methods:**

The authors used an exploratory mixed methodology starting with curriculum mapping of the medical curriculum, followed by key informant interviews with the University of Notre Dame, School of Medicine academics (n = 6) and alumni (n = 5) on teaching/learning and practice of health advocacy.  The final stage consisted of a cross-sectional survey on teaching/learning health advocacy among third and fourth (final) year medical students (N = 195).

**Results:**

The medical curriculum contained no explicit learning objectives on health advocacy. Key informant interviews demonstrated an appreciation of health advocacy and its importance in the medical curriculum but a deficit in explicit and practical ‘hands-on’ teaching. Survey response rate was 47% (n = 92). A majority of students (76%, n = 70) had heard of health advocacy, with this being more likely among third (92%, n = 33) compared with fourth-year students (67%, n = 37) (Fisher’s Exact Test χ^2 ^(2, N = 91) = 7.311, p = 0.02). Students reported having opportunities to observe (76%, n = 70) and practise health advocacy (50%, n = 46) in the curriculum.

**Conclusions:**

Students and medical graduates demonstrated sound recognition of the term health advocacy. Deficits identified in the curriculum include lack of explicit learning objectives and “hands-on” learning opportunities in health advocacy.

## Introduction

Health advocacy (HA), defined by the CanMEDS framework as “physicians responsibly using their expertise and influence to advance the health and well-being of individual patients, communities, and populations,” has emerged as an essential skill for medical practitioners.^1^ HA applies to multiple contexts within medicine including patient education, assistance with navigating healthcare resources, leadership and social activism.^2^ HA is now recognised as a skill that ought to be developed in medical students to foster socially responsible doctors.^3^^,^^4^  Regulatory bodies including the Australian Medical Council (AMC) formally adopted HA in their Standards for Assessment and Accreditation of Primary Medical Programs in 2012.^5^ One of the AMC’s four key domains for medical graduate outcomes is “The medical graduate as a health advocate”.^5^ Graduates are expected to “communicate effectively in wider roles including health advocacy, teaching, assessing and appraising” and to have an understanding of the impact of social determinants on health.^5^ The intern outcome statements published by the AMC also emphasise that interns must apply their knowledge of the social, economic and cultural factors that impact population health.^6^^,^^7^ Despite these requirements, a previous systematic review in 2014 showed that there is no published literature on the best practice evidence for teaching HA to medical students.^7^ North American medical schools have begun introducing HA into the curricula in a variety of different ways in the last two decades.^8^^-^^14^ However, it remains an underexplored area of medical education.

Experiential learning and integration of theoretical knowledge with practical experience through clerkships has been long adopted for clinical skills training in medical schools worldwide. Potentially, there could be cases of undocumented experiential learning in HA, though only a few such instances were reported in the medical journals.  Nonetheless, adopting HA experiential learning in the context of advocacy is seldom pursued in the current medical school teaching agenda except for a few published articles. In 2004, the University of Rochester Medical Centre made their Community Health Improvement Clerkship (CHIC) compulsory for fourth-year undergraduate students.^9^ Students were required, in this four-week course, to implement a community health project in conjunction with local agencies. Medical students were encouraged to think about issues such as smoking, homelessness, chronic illness, and obesity at a broader population level. It involved tasks such as health promotion activities and collaboration with community health physicians to create “personalized educational interventions” for socio-disadvantaged population groups affected by obesity.^9^ Students were closely mentored, community participants gave informed consent and there was infrastructure in the medical school and local agencies to sustain the programs so they could be continued and further developed by future students. An evaluation (four weeks after project completion) showed that the majority (94%) of students who undertook the “hands-on” CHIC reported that the course had impacted their future career favourably.^9^ The study lacked longitudinal follow-up to assess the influence of the program long-term. Nonetheless, additional studies have demonstrated that optimal training in advocacy requires practical “hands-on” experience.^8^^,^^11^^,^^12^

Studies in North American medical schools suggests that exposure to HA teaching programs through “hands-on” community involvement is associated with greater knowledge in community health issues and improved social attitudes of students towards these issues.^8^^,^^9^ Dharamsi and colleagues conducted an explorative study of medical students’ experiences in international service-learning.^13^ This study demonstrated that while it is not known how to best train medical students in HA, experiential learning and critical reflection is central to developing the necessary knowledge and skills to become health advocates.^13^

In addition, the University of Miami Miller School of Medicine assessed the impact of a “hands-on” project with community-based organisations (CBOs) in 2014.^8^ The curriculum included two didactic lectures, followed by student-led advocacy projects over two years on a health issue of their choice. The final proposal of an evidence-based intervention designed by the students was presented to the CBO at the end of the two years. This curriculum was undertaken at the rural campus and was compared to the control group at the city campus where this project was not undertaken. The results of the survey demonstrated high self-reported skills and knowledge in how to undertake a project in HA and greater understanding of the community’s health needs, compared to the comparison city cohort.^8^ 

Health advocacy includes engagement with broader networks outside of medicine including politicians. The medical schools of the University of Alberta and the University of Calgary (2011) conducted a two-day intensive course in HA, with one day of HA training and the second day involving smaller groups practising pitching a health issue to local politicians employing the HA skills they had learnt.^11^ It proved an efficient exercise given the time limitations of an already full undergraduate curriculum. Furthermore, an American study in 2012 looked at the impact of a half-day advocacy experience by attending the National Advocacy Day as part of the American Medical Student Association’s convention in Washington, D.C. Pre- and post- surveys were used to assess the pre-medical and medical students’ views and knowledge of HA.^12^ The experience demonstrated an improved understanding of how health policy influences the health of individuals and populations, as demonstrated through Likert scale scores and increased likelihood to be able to seek ways to be health advocates in their future medical training.^12^ Based on a series of provided statements, the lowest Likert score in knowledge recorded by students was in response to: “My medical school curriculum has provided me with sufficient health legislative advocacy training” with a mean score of 1.99 post-experience survey.^12^ This gap in medical education curriculum was further highlighted in a Canadian survey (2013) of medical residents, which demonstrated three-quarters of resident trainees had never had any health policy training.^14^

Overarching, the dominance of a biomedical approach to medical practice is likely to have contributed to HA being an underexplored and unrecognised area of the medical education.^15^ Political arguments of sceptics surrounding the subject suggest that advocacy training lies outside the realm of medicine, that “the traditional norms of scholarships: accuracy, objectivity and truth” should not be “entangled” in political activity.^16^ Views such as these may covertly and unintentionally influence the design of medical curricula, despite “The medical graduate as a health advocate” being one of the AMC’s four key domains for medical graduate outcomes.^5^ In addition, the teaching of HA is likely limited by uncertainty among academics as to how this is best taught, given a lack of literature on the subject.^13^

Although governing bodies such as the AMC support and mandate HA teaching in medical schools, it is unclear as to whether HA is actually being formally and informally taught in Australian medical schools. If HA is being taught, the methods by which it is being taught have not previously been explored or published in the literature. Therefore, the objectives of this research project are to explore the teaching and learning of HA in an Australian medical school. The aim of the study was to answer the following exploratory question: Are graduates of the University of Notre Dame, Fremantle (UNDF) School of Medicine (SoM) educated and skilled in health advocacy for the benefit of their future practice with patients and the wider community?

## Methods

### Study design

We conducted the study with a three-phase exploratory sequential mixed-methods design; 1) curriculum mapping, 2) qualitative analysis of key informant interviews with academics and medical graduates, and 3) a quantitative survey of medical students. Sequential exploratory mixed methods design approach utilises the findings of a qualitative data collection technique (semi-structured interviews) to inform a quantitative final phase (a descriptive survey).^17^ In a sequential mixed methods design study, each subsequent data collection is reliant on the findings of the preceding phase to inform the direction of the research. The Human Research Ethics Committee of the University of Notre Dame Fremantle approved this research. Ethical considerations were made to ensure participating students, staff and graduates were provided with an information sheet detailing the study and opt-out of reminder emails if they did not wish to participate.

### First phase: curriculum mapping

#### Instrument

We used the UNDF SoM electronic curriculum database to search the explicit key terms “health advocacy” and “advocat*.” We also searched for implicit key terms related to HA, including “promot*,” “prevent*,” “social determinants,” “activis*,” “empower*” and “community health.” The symbol * refers to all words searched with that prefix. This database contains the AMC Institution Outcomes, Bachelor of Medicine/Bachelor of Surgery (MBBS) Outcomes, and the UNDF SoM unit goals, broad learning objectives, student learning objectives and resources.

### Data analysis

Data were collected by author AD, using Microsoft Xcel, by recording the number of times the key terms appeared within the curriculum and where they occurred in the curriculum.

### Second phase: key informant interviews with academics and medical graduates

#### Instrument

The interview schedule comprised six open-ended questions, which is appropriate for the semi-structured interview approach(Appendix 1).   Interviews took place at the UNDF Fremantle campus and were audio-recorded. The interviews were an average of thirty minutes in length and were transcribed and analysed using thematic analysis and independently cross-checked by CB.

#### Participants

AD interviewed eleven informants using a purposeful sampling technique comprising six UNDF SoM academics and five UNDF medical graduates. Purposeful sampling is a non-probability qualitative sampling technique, which seeks to select those with the greatest amount of in-depth information around a specific topic, event or phenomenon.^18^ Selection criterion for selecting the academic informants was based on their contribution to the development, updating and responsibility for delivery of the UNDF medical curriculum.  The academics selected were the Dean, Associate Dean of Aboriginal Health, Head of the University’s Core Curriculum in Medicine (UNDF students in all courses undertake compulsory units of Theology, Philosophy, and Ethics), and domain chairs of Communication and Clinical Practice (CCP), Population and Preventive Health (PPH) and Personal and Professional Development (PPD). CCP is the teaching of clinical and communication skills. PPH encompasses epidemiology and public health, whilst PPD covers professionalism and the fostering of self-reflective clinical practice.

Medical graduate informants were purposively selected based on their prior involvement as office bearers in the student representative body Medical Student Association of Notre Dame (MSAND) to ensure that informants would be willing and able to discuss their views on the teaching and learning of HA. All previous MSAND medical graduate representatives from 2014, 2013 and 2012 were invited to participate (N=16); five (three females and two males) responded and were interviewed.

### Data analysis

Inductive Thematic Analysis (TA) is a method for identifying and analysing patterns of meaning in a dataset.^18^ This approach incorporated four parts: naïve reading, comprehensive understanding and interpretation, structured thematic analysis and matrix coding.^19^ Firstly, transcriptions were read repeatedly to ensure familiarity with the text, followed by open coding to all transcriptions to derive themes by comparing across all transcripts.  Subsequently, the transcripts were coded line by line to determine if any subthemes emerged.  As a form of validation for the findings, a selection of transcripts were also independently read and coded by three other members of the team. The team then met to discuss common themes and reach agreement regarding coding structure.

### Third phase: Survey of medical students

#### Instrument

Results of the curriculum mapping and the thematic analysis facilitated the online questionnaire design. This consisted of 22 items including seven demographic questions. This was followed by seven Likert-scale questions to measure from ‘strongly disagree’ to ‘strongly agree’ the student’s attitudes, values and confidence in understanding HA’s role in medicine.  On a subsequent page, students were then provided with our study’s definition of HA, “the active pursuit of promoting positive change to benefit the health outcomes of an individual or population group.” In providing our definition, we ensured the validity of responses to the following six questions. These six questions included three trichotomous questions asking students if they had learnt, observed or had the opportunity to practice HA in the medical curriculum, with options to answer ‘yes,’ ‘no’ or ‘unsure.’ Each question was followed by the option to provide up to three open-end responses of where this occurred in their training. Participants were required to provide at least one example. We pretested the survey on eight individuals two weeks prior to the survey was disseminated. Technical errors were corrected, and several questions were re-written.

#### Participants 

All 195 (81 males and 114 females) medical students at UNDF in third (113) and fourth (82) year were invited to complete the survey within a three-week period during which the online survey was open.

#### Data analysis

The survey data was collected through an online survey instrument and analysed with SPSS 23.0 (IBM, United States of America). Demographic characteristics of respondents were independent variables, and the dependent variable was whether the student had heard of the term HA. ‘Unsure’ responses to the question, ‘Have you heard of the term HA?’ were combined with the ‘no’ responses to create a binary outcome variable. This is because we considered those students who were ‘unsure,’ were unlikely to be confident with the term, and therefore closer to a ‘no’ then a ‘yes.’ The dependent binary variables were: ‘have heard of the term HA’ = 1 and ‘have not or unsure if heard of the term HA’ = 0.

Associations between the dependent and independent variables were explored using chi-square analysis. The null hypothesis was that there was no association between whether a student had heard of the term HA and the various demographic variables. Where more than 20% of the expected frequencies were less than five, data was collapsed, and we used a Fisher’s exact test to calculate the p-value.

Following this, we performed logistic regression analysis to determine if there was any particular demographic variable that was influencing whether a student was more likely to have heard of the term HA.

## Results

### First phase: curriculum mapping

The explicit key terms search of “health advocacy” and “advocat*” within the SoM database yielded four results. HA appeared twice at the overarching level of the AMC Institutional outcomes “the medical graduate as a health advocate” and MBBS level: “a commitment to advocate for, and to facilitate, access to health care for members of underserved and marginalised populations.” HA was also apparent in PPH teaching resources, including PPH lectures and panel discussions involving expert patients and  representatives of community-based organisations.  However, HA did not appear in the UNDF unit goals, broad or student learning objectives.

### Second phase: key informant interviews with academics and medical graduates

Four key themes emerged from the interviews with academic staff (A1-6) and graduates (G1-6).

#### Theme 1: Sound understanding of ‘health advocacy’

Most participants were able to articulate the meaning of the term ‘health advocacy’ (HA) around representing the needs of those who need it most. Furthermore, participants explained HA extends beyond the traditional role of the doctor as clinician, into a leadership role as a socially responsible contributor in society. This involves helping patients to address socio-cultural and economic factors that impact on their health. Two staff interviewees explained:

“It’s about the role of advocating for an issue related to health for people who actually can’t do it for themselves”. (A4, female)

And,

“It might also extend a bit further than that because they might need our help to get adequate housing or social security payments or perhaps liaising with an employer, so they can have appropriate employment if they’re injured, etc.” (A6, female)

This theme was consistent among the medical graduate interviewees who believed that being a health advocate involves ensuring each patient has access to the care they need, particularly for patients recognised as needing advocacy most.

“[It’s about] going in to bat for people who don’t have the best access to health care…” (G5, male)

In terms of perceived importance of HA, all interviewees said it was essential for medical doctors to be involved in HA as influential role models and leaders in society. One academic explained the positive impact that doctors have when they use their occupational status to publicly support disadvantaged groups in society. A second academic reiterated this point in stating:

“…It’s something I try to stress to my students…that they need to see their role as not simply the everyday interactions they have with patients but [that] also they also have a public role…” (A5, male)

Graduates reiterated the important role of HA and leadership in medical practice as being “an ambassador” (G3, female) and ensuring ongoing patients’ maintain “good health in the long term” (G2, male).

#### Theme 2: Health advocacy teaching predominantly in the non-clinical curriculum

All five graduates reported being exposed well to the importance of HA as students. Key examples specified by graduates about where HA was taught, were: interactive panel discussions involving expert patients and representatives of community-based organisations; rural non-clinical placements in pre-clinical years; and the fourth-year clinical audit project. One graduate quoted:

“…Especially in the symposiums ... I remember hearing from the Parkinson’s group and have vivid memories of some of the community people about what they’ve got and [how] they’re trying to improve the health of people with Parkinson’s in the community, [and]…from the sex workers, from all kinds of different community groups”.  (G5, male)

The majority of academics interviewed believed non-clinical rural placements were highly important in developing the students’ skills in advocacy. Placements included a four-day community placement in a rural town in first year and a ten-day placement in second year in the Kimberley region of Western Australia.^20^ One academic explained in relation to the students’ preparation for the Kimberley placement:

“In [preparing for] the rural health [placement], they are doing debates and having to put forward a position; it’s a skill development actually, in thinking through those things”.  (A4, female)

While the majority of graduates reported that knowledge in HA was learnt on-campus through coursework, they also reported developing their HA skills in their former careers or in leadership roles in extra-curricular student organisations:

“…Being involved with MSAND and AMSA [Australian Medical Students’ Association] probably encouraged my [advocacy] skills in clinical years”. (G1, female)

#### Theme 3: Barriers and enablers to learning health advocacy

There were a number of perceived barriers and enablers to HA learning that were identified by both the graduates and the academic participants. Key enablers were identified as the focus on the mission of the SoM, which educated students in a way that values social justice; and that the UNDF medical course is graduate-entry, so students may have background work experience in advocacy. A third enabler was the presence of passionate role models as teachers and leaders within the UNDF SoM. Certain teachers strongly promote the School’s mission and communicated the importance of having medical doctors who are health advocates. Graduates also highlighted the importance of the individual student’s personal interest and motivation for skill development in HA.

“I think at the end of the day, some people choose not to be engaging with that, and hopefully a little bit has got through and eventually they’ll be like, that’s part of their job”. (G1, female)

And,

“…If you choose to engage you will learn a lot from it”. (G5, male)

Conversely, barriers to HA learning were perceived to be the implicit (hidden) nature of HA in the curriculum along with the belief among academics that students perceived the curriculum content covered in population health as not as important as the scientific and clinical knowledge components.

“It could well be missing because we don’t have labels or learning objectives or obvious assessment questions in exams which are labelled as ‘health advocacy’.” (A1, female)

This was corroborated by another academic who stated HA is definitely implicitly taught in the Aboriginal health curriculum:

“We provide engagement with community where the students get to learn about the realities of people‘s lives and what’s going on in community, and so [they] get exposed to areas of potential action that they could engage with”. (A2, male)

Both academics and graduates described one additional barrier: that doctors may not feel comfortable ‘going the extra mile’ in advocating for their patients’ needs, given the time and pressure constraints on clinical placement. As one student explained,

“…We were really rapidly moving through patients, but we didn’t really get the opportunity to evaluate people’s social situations”. (G1, female)

#### Theme 4: Curricular opportunities for improvement largely skill-based

One strong theme that emerged was that graduates felt their knowledge was adequate, but their practical HA skills were lacking. One graduate discussed a lack of opportunity to develop skills in HA as a medical student on clinical placements in hospitals:

“I found that our role was a fairly passive one… taking virtually little part in actually managing the patients… We were always kind of worried on giving advice or promoting health or being advocates for health because we didn’t want to [over] step the boundary and maybe give the wrong information or do the wrong thing”. (G2, female)

It was suggested by one graduate that including more activities to develop argument formation would greatly assist in building skills in terms of HA.

#### Third phase: Survey of medical students

Ninety-two of one-hundred and ninety-five students (47%) completed the online survey. The response rate among third and fourth-year students was 39% and 61% respectively, with 39% male and 61% female, mean age of 29.6 years (Table 1).

**Table 1:** Demographic characteristics of medical student survey respondents and medical students invited to participate in the UNDF survey in 2015

Characteristic		Survey respondents (n=92)	Students invited to complete the survey (n=195)
		n (%)	n (%)
Gender	Male	36 (39)	81 (42)
	Female	56 (61)	114 (58)
Age (years)	Mean ± SD	29.6 ± 4.4	30.0 ± 4.9
Year level	Third Year	36 (39)	112 (57)
	Fourth (Final) Year	56 (61)	83 (43)
Current or previous Rural Clinical School^*^ student	Yes	29 (32)	54 (28)
	No	63 (68)	141 (72)
Duration of volunteer experience prior to applying for medical school	Never volunteered	11 (12)	Data not available^**^
	<1 year	28 (31)	
	>1 year	53 (57)	
Time spent living in rural or remote Australia prior to medical school	Never lived rurally	47 (51)	Data not available^**^
	<1 year	11 (12)	
	>1 year	34 (37)	
Highest level of education completed	Bachelor’s Degree ± Honours Graduate	68 (77)	Data not available^**^
	Diploma/Master’s Degree	19 (21)	
	Doctorate	2 (2)	

^*^Students are selected via a competitive application process to undertake the third year of the course in one of 13 Western Australian rural towns.^25^

^**^Data not available due to the university’s privacy regulations.

The responses to the six Likert-scale questions confirmed 98% of respondents had a very high level of understanding of the social determinants of health. All stated it is essential to be knowledgeable about the social determinants of health to be a good doctor.

The majority of respondents (n = 70, 76%) reported they had heard of the term HA while 14% (n= 13) had not and 9% (n = 8) were uncertain. A larger proportion of third (92%, n=33) than fourth (67%, n = 37) year students (Fisher’s two-sided χ^2^ (2, N = 91) = 7.311, p = 0.02) reported having heard of HA. There were no other statistically significant associations between the other demographic variables listed in Table 1 and having heard of HA.

Participants who had heard of HA were asked to identify where they had heard of HA. The most common responses were SoM lectures (n=12, 16%); the PPH curriculum (n=10, 13%), including the fourth-year clinical audit project; the PPD curriculum (n=8, 11%); prior university degrees (n=7, 9%); the media (n=7, 9%); clinical placement (n=6, 8%); CanMEDS framework (n=5, 7%) and other sources (n =20, 27%) including medical literature, politics, other medical students, nursing and allied health staff.

A majority (n = 70, 76%) of survey participants reported they had the opportunity to ‘observe HA’ in medical school and 73% (n= 67) reported being ‘taught about HA’. However, only 50% (n= 46) of participants stated they had the opportunity to ‘practice HA’ as a medical student.

## Discussion

Our exploratory sequential mixed-methods approach aimed to determine whether graduates of UNDF SoM are educated and skilled in HA for their future practice. We believe our study is the first to provide robust evidence on current teaching/learning experiences in HA in an Australian medical school.

Curriculum mapping confirmed that HA was only mentioned at the higher levels of MBBS and AMC institutional outcomes (not in broad and specific course learning objectives accessible to students). There was minimal explicit HA teaching evident in the UNDF medical curriculum. However, the interview and survey data established a range of teaching/learning activities in the course where students learned about HA, without necessarily labelling it explicitly as ‘HA’. The examples provided were largely experiential learning opportunities, such as rural non-clinical placements and the interactive panel discussions involving expert patients and representatives of community-based organisations. These examples are consistent with North American studies suggesting that it is largely through “community health improvement clerkships”,^9^ research projects,^10^ health policy workshops^11^ and opportunities to collaborate with politicians on local health issues^11^ where students learn to be health advocates.

The response rate was 47% in our survey, and the age and gender distribution of respondents was comparable with that of all students eligible to participate (Table 1). Three-quarters of students were familiar with the term HA. The significant difference in recognition of the term HA between the year three and year four groups was unexpected and may be in part due to the difference in response rate between the two cohorts: 39% and 61%, respectively. This may have resulted in an unequal distribution of unknown confounding factors between the two-year groups. One-quarter of the respondents had never previously heard of the term HA. This identifies a gap in explicit learning/teaching of HA which is corroborated with the findings of the curriculum mapping and the interviews. In Canada, the University of Toronto has recognised this gap in medical education and implemented a two-year health policy curriculum directed at family medicine residents in training.^14^ The results demonstrated a 25% increase in self-reported understanding of the Canadian health care system and overall positive evaluation demonstrating that medical students and residents are keen for an education in health policy.^14^ Having explicit learning objectives and teaching/learning modules could address this gap.

Despite this large group who were unfamiliar with HA, the majority of students felt confident in their understanding of the social determinants of health. This is consistent with the results of a Canadian survey of seventy-six medical residents, where the majority felt HA was very important and were able to identify the social health determinants for a patient beyond their biomedical needs.^21^ However, again, this study demonstrated that only one-quarter of surveyed medical residents were actively engaged in HA activities.^21^

Despite the majority reporting learning and observing HA, only half of surveyed students had the opportunity to practice HA during the course. This indicates an opportunity for improving experiential learning in HA, for example through international^13^ or community service projects.^8^ While evidence is limited, the results indicate that specific HA “activities”^9^ improves knowledge of health policy and it fosters a compassionate awareness among training doctors for how existing social, cultural and economic disadvantages influence health status.^13^

### Limitations

One potential limitation of the study is information bias. The PPH domain staff interviewee had extensive background knowledge of HA and co-supervised the project. A sensitivity analysis performed by removing this transcript did not alter the findings, so this transcript was included in the analysis.

The small sample size of interviewees, both staff and alumni, is another limitation in terms of broader generalisability. As a strategy to increase the response rates, we purposively sampled medical graduates who had been MSAND office bearers, which could introduce selection bias.  The lower response rates of year three students may have introduced a selection bias to our understanding of implicit teaching/learning of HA. However, the higher response rate of year four (final year) students provides a more representative sample mitigating this issue.  Furthermore, the results of this study may not be applicable to other medical schools as each school has a unique curriculum, individual program entry requirements, and mission for their School of Medicine.

### Future directions

These research findings have prompted the UNDF SoM to introduce explicit HA learning objectives and additional teaching and learning activities to help students achieve these learning objectives. This includes an interactive HA workshop and “The Game of Greater Good” (a simulation of health resource allocation in which some students take on the role of community advocates who seek to influence health policy and funding decisions).^22^ Furthermore, a chapter on HA has been written and included in the third-year population and preventive health workbook.

Future developments of this research project include determining the best practice methods for teaching and learning HA. This requires evaluation of the post-graduation outcomes of HA teaching in medical school on alumni’s career direction, clinical practice, and professional development.

## Conclusions

This study has demonstrated that some, but not all medical graduates of UNDF, are adequately prepared to be health advocates. This was evident given the proportion of survey respondents who were unfamiliar with HA, the lack of explicit HA learning objectives and labelled HA activities. HA has been recognised as a core competency by the AMC for all medical graduates but there exists no formal framework or evidence for how this is best taught. Despite the AMC’s requirements, the absence of HA in broad and specific course learning objectives accessible to students was not an impediment to the school’s accreditation.

In conclusion, it is the responsibility of universities to ensure medical students meet these competencies. The absence of explicit HA learning objectives is a component of the medical curriculum that ought to be addressed. It is a subject of great significance for medical educators given the increasing burden of preventable chronic diseases worldwide. With increasing complexities to the health care system, rising health care costs and growing disease burden in socio-disadvantaged groups; the implementation and evaluation of explicit HA advocacy teaching in medical curricula should be a priority. Providing affordable, cost-effective and personalised whole patient-centred care for Australian patients will require future doctors who are proactive problem-solvers, who can advocate for their patients and their community.

### Acknowledgments

The authors thank Dr. R. Oehmen and Prof. M. Bulsara (UNDF) for their advice on statistical analysis. Thank-you to Verity Ho for transcribing the interviews. Thank-you to the staff and alumni interviewees and the medical students for participating in the study. Lastly, thank-you to the Public Health Advocacy Institute of Western Australia (PHAIWA) which expressed written enthusiasm for this research.

### Conflict of Interest

The authors declare that they have no conflict of interest.

## Supplementary material

Supplementary file 1
Appendix 1. Interview Guide questions used in Phase Two of the key informant interviews
