# Supplementary material for: The teaching and learning of health advocacy in an Australian medical school
Source: Int J Med Educ. 2018 Jan 31;9:26–34. doi: 10.5116/ijme.5a4b.6a15 (PMC5834824; doi:10.5116/ijme.5a4b.6a15)
Supplement: Supplementary file 1 — Appendix 1. Interview Guide questions used in Phase Two of the key informant interviews [file ijme-9-26-S1.pdf]

Appendix 1

Interview Guide questions used in Phase Two of the key informant interviews

Academic staff interviews

1. Have you heard of the term ‘health advocacy’?
2. Do you think it is important for a medical doctor to be involved in health advocacy?
3. If you think about the current curriculum…what aspects, if any, do you think develop the attitudes, knowledge, and skills required for effective health advocacy?
4. Does your particular domain provide knowledge and/or skills in health advocacy to the medical students within the current curriculum?*
5. What do you think are (a) the barriers and (b) enablers to medical students acquiring the attitudes, knowledge, and skills required for effective health advocacy?
6. Describe the role that you believe the learning of public health policy and political advocacy should have in the teaching of medical students at UNDF.

*Question 4 was altered for the Dean of the School of Medicine to “Do any particular domains and/or the core curriculum provide knowledge or skills in health advocacy to the medical students within the current curriculum?”

Medical graduate interviews

1. When you hear the term ‘health advocate’, what does this mean to you?
2. If you reflect back on your medical career to date, both as a student and professional, are you able to share perhaps one or two examples of the best and worst experiences you’ve experienced in health advocacy?
3. Could you please outline the knowledge and skills that were critical in developing your own skills in health advocacy?
4. To what extent do you consider your current knowledge and skills in health advocacy adequate for effective health advocacy?
5. To what extent did your medical school education prepare you to be an effective health advocate for your patients?

Do you think that Notre Dame medical school could have better prepared you to be an effective health advocate or was the preparation adequate for you?
